# Carbon Dioxide Release During Photosynthesis: Connecting Gas Exchange Behavior With Biochemistry

**DOI:** 10.1111/pce.70216

**Published:** 2025-09-29

**Authors:** Thomas D. Sharkey, Yuan Xu

**Affiliations:** ^1^ Department of Biochemistry Michigan State University East Lansing Michigan USA; ^2^ MSU‐DOE Plant Research Laboratory Michigan State University East Lansing Michigan USA; ^3^ MSU Plant Resilience Institute Michigan State University East Lansing Michigan USA

**Keywords:** carbon13 labeling, gas exchange, oxidative pentose phosphate pathway, photosynthesis, respiration in the light

## Abstract

During photosynthesis, CO₂ uptake is counterbalanced by concurrent CO₂‐releasing processes, complicating the interpretation of gas exchange measurements. While photorespiration accounts for a significant portion of this CO₂ release, emerging evidence indicates that there are additional metabolic pathways that release CO_2_ during photosynthesis. This metabolism—termed day respiration (often *R*_*d*_) or respiration in the light (*R*_*L*_)—is now recognized as an independent and significant source of CO_2_ emission during photosynthesis. Here we revisit classical models of photosynthesis and incorporate new insights from isotopic labeling and metabolic flux analysis (MFA) to investigate the biochemical basis of *R*_*L*_. We identified the cytosolic glucose‐6‐phosphate (G6P) shunt through the oxidative pentose phosphate pathway (OPPP) as the predominant contributor to *R*_*L*_. This shunt explains some long‐standing anomalies in Calvin‐Benson‐Bassham (CBB) cycle labeling. Under non‐stressed conditions, *R*_*L*_ remains stable across varying CO₂ concentrations and light intensities. Under heat stress, *R*_*L*_ shifts toward a plastidial source. Together, these findings resolve longstanding questions about carbon flux during photosynthesis and improve our understanding of *R*_*L*_ by explaining its metabolic origin, physiological significance in carbon balance during photosynthesis, and regulation under varying environmental conditions.

## Summary statement

This review clarifies the metabolic origin of light‐driven CO₂ release, highlighting the dominant role of the cytosolic G6P shunt via the oxidative pentose phosphate pathway, resolving long‐standing anomalies in carbon labeling and advancing our understanding of plant carbon balance.

## 1 Introduction and History

Loss of CO_2_ during simultaneous CO_2_ uptake up by a C_3_ leaf was termed by Otto Warburg a “nightmare oppressing all who are concerned with the exact measurement of photosynthesis, and various attempts have been made to bring it to light” [found on page 569 of Rabinowitch (1945)]. Knowledge of light‐dependent processes other than core carboxylation reactions was needed to address, among other things, the controversy surrounding the measurement of the minimum number of photons required to fix one CO_2_ molecule (Nickelsen and Govindjee 2011). Over the past 50 years, advances have linked gas exchange behavior to the underlying biochemistry of photosynthesis. The “nightmare” has since become manageable, as we now recognize specific processes that release CO₂ and partially reverse photosynthetic CO₂ uptake. Most of this counter flow of CO_2_ is understood as photorespiration. This results in a post‐illumination burst of CO_2_ (Decker 1955; Sharkey 1985; Gregory et al. 2024). The post‐illumination burst is actually composed of several processes. There is post‐illumination CO_2_ fixation and this has been used as a measure of the amount of RuBP present when the light is turned off (Laisk et al. 1984). Glycine decarboxylation is thought to occur more slowly and so outlasts post‐illumination CO_2_ uptake (and in any case post‐illumination CO_2_ fixation will also supply additional glycine under photorespiratory conditions). Based on effects of factors that reflect photorespiration, which will result in different pool sizes of active glycine (Fu et al. 2023), glycine decarboxylation is likely the most important contributor to the post‐illumination burst. Yet another source of CO_2_ burst after turning off the light results from the acidification of the chloroplast. This can result in bicarbonate conversion to CO_2_, which can contribute to post‐illumination fluxes.

The first step of photorespiration was identified by (Bowes et al. 1971; Ogren and Bowes 1971), who demonstrated that photorespiration results from the oxygenation of ribulose 1,5‐bisphosphate (RuBP) at rubisco, rather than carboxylation. Subsequent analysis of Arabidopsis mutant lines confirmed the key steps in photorespiration (Somerville 2001), highlighting Arabidopsis as a powerful tool for plant metabolism studies.

## 2 Effects of Photorespiration on Gas Exchange Behavior

Recognition that there are one or more sources of CO_2_ release during photosynthesis uptake led to terminology of gross or true photosynthesis versus net photosynthesis. Today it is more common to speak of the velocity of carboxylation (*v*_c_) and net photosynthesis (*A*). Photorespiration is given by the velocity of oxygenation (*v*_o_) and can affect gas exchange behavior in three ways:

1. CO_2_ is released by decarboxylation of glycine (Eisenhut et al. 2019),
2. the metabolism of photorespiration consumes energy that might otherwise be used for carbon assimilation (Walker et al. 2024), and
3. reduction of the efficiency of rubisco by acting as a competitive inhibitor of carboxylation (Bathellier et al. 2020).

Photorespiration involves oxygen uptake and CO_2_ release like animal respiration but consumes energy rather than producing it. Nevertheless, it is called photo*respiration*.

Once oxygenation by rubisco was known it was possible to model it to compare biochemical understanding with gas exchange behavior. Laing et al. (1974) published the following equation:

(1) $$A=v_{c}-t{∙v}_{o}$$

where *A* (they used *P*_*n*_ for net photosynthesis) is the net rate of CO_2_ uptake and *t* is the number of CO_2_ molecules lost per oxygenation event. (We use lower case *v*, as is standard in biochemistry, to help distinguish this rate from maximum velocity, e.g. *v*_*C*_ versu*s V*_max_. This capitalization convention is similar to that of *K*_*m*_ *vs. k*_cat_). Initially *t* was taken to be 0.25, but with improved understanding of glycolate metabolism (Tolbert 1971) this was changed to 0.5 by Keck and Ogren (1976). In other words, one CO_2_ is released for every two oxygenation events. Photorespiration accounts for most of the CO_2_ evolution during photosynthesis. However, it was not clear whether other CO_2_‐releasing metabolism was occurring along with photorespiration at rates that might affect gas exchange behavior. When Farquhar et al. (1980) published on the relationship between gas exchange behavior of C_3_ leaves and the underlying metabolism, they allowed for other CO_2_‐releasing reactions. This is often considered to be residual tricarboxylic acid (TCA) cycle activity. This was called dark respiration in the light, *R*_*d*_. They also settled on *t* to be 0.5. So, by 1980 the equation was (Farquhar et al. 1980):

(2) $$A=v_{c}-0.5{∙v}_{o}-R_{d}.$$

**Figure 1:**
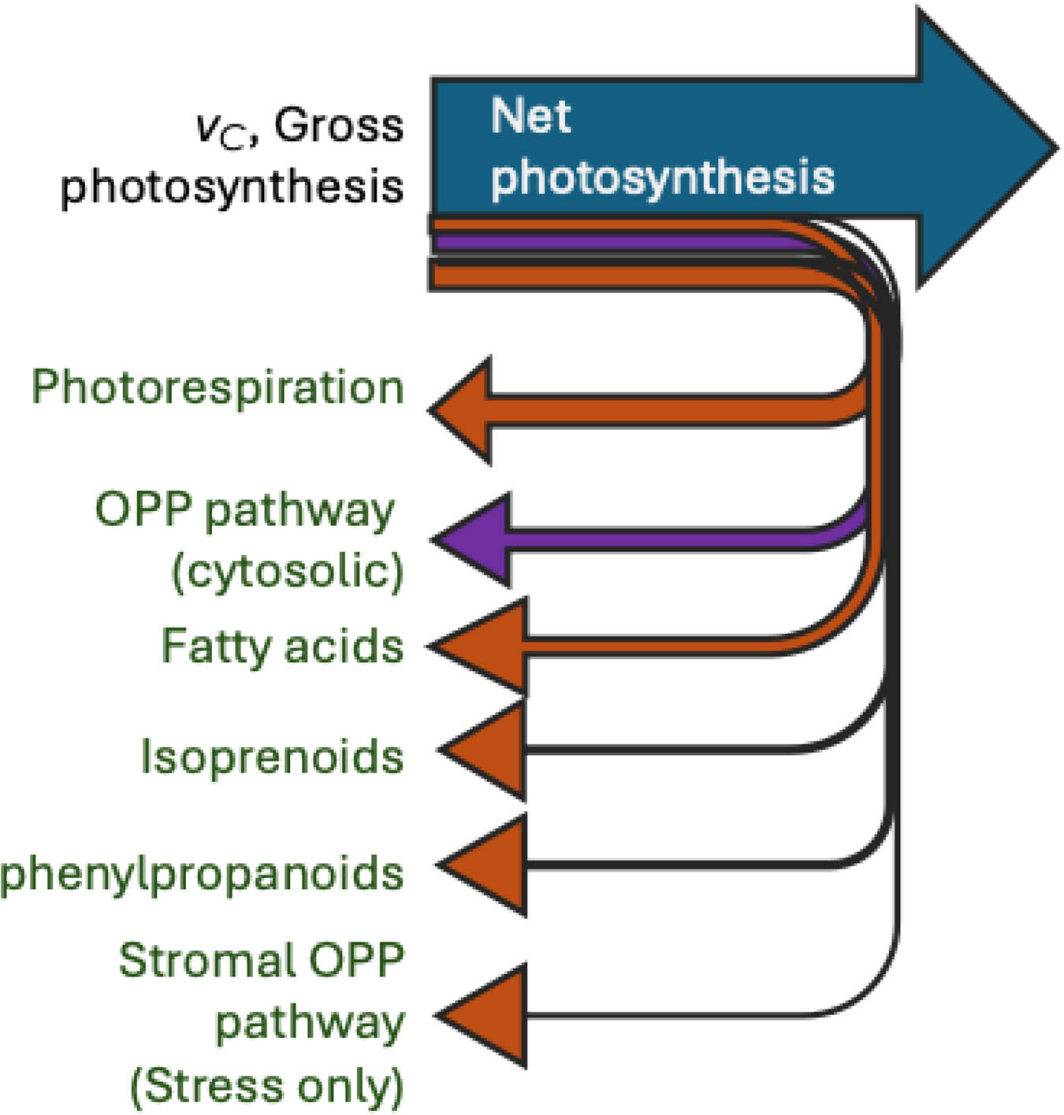
Net photosynthesis is determined by the velocity of carboxylation minus CO_2_ lost to photorespiration and other CO_2_‐releasing metabolism. The arrows indicate the direction and relative magnitude of carbon flow. The purple arrow is the metabolism we suggest is responsible for most of *R*_*L*_.

Equation 2 is the starting point for many, many photosynthesis models and is shown pictorially in Figure 1. This equation can be used to connect gas exchange behavior with biochemistry.

An important contribution of Farquhar et al. (1980) was the recognition that the interaction between rubisco and its substrate, RuBP, followed kinetics of a tight binding inhibitor (or tight binding substrate, as in this case) (Morrison 1969; Farquhar 1979). In biology we often think of control being spread throughout the system but, in the case of rubisco and RuBP regeneration, it is an either‐or situation. Gas exchange will reflect *either* RuBP‐saturated kinetics (the rubisco limitation) *or* RuBP limiting kinetics (*J*‐limited). The familiar Michaelis Menton kinetics apply only when the substrate binding affinity is much greater than the binding site concentration (Sharkey 2023). The Michaelis Menton kinetics are a special case that occurs when the concentration of substrate is much greater than the concentration of binding sites. This behavior is modeled with the following equation (Morrison 1969):

(3) $$v=\frac{b-\sqrt{b^{2}-4∙\left[E_{T}\right]∙\left[S_{T}\right]}}{2∙\left[E_{T}\right]}$$

where b=$\left[E_{T}\right]∙\left[S_{T}\right]∙K_{M}$, [*E*_*T*_] = total amount of enzyme, and [*S*_*T*_] = total amount of substrate.

**Figure 2:**
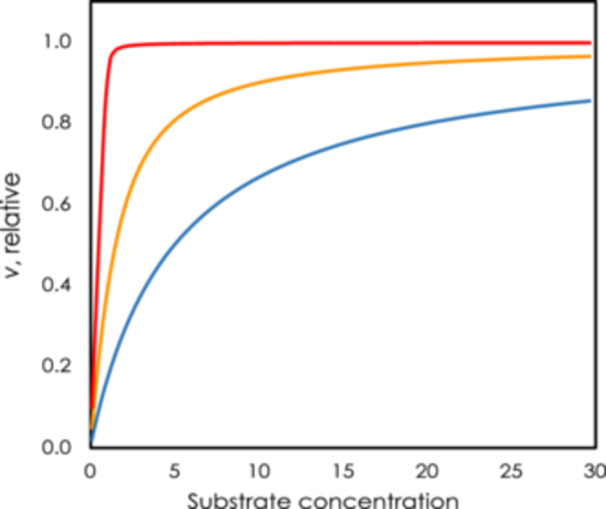
Enzyme kinetics according to Equation 3 of the text. The blue line is for the condition where substrate the *K*_*m*_ is five times greater than enzyme sites, orange is where sites and *K*_*m*_ are equal and the red line is for the condition where the concentration of sites is 100 times less than the *K*_*m*_, the situation that applies to rubisco and RuBP (Modified from Sharkey (2023). [Color figure can be viewed at wileyonlinelibrary.com]

When the concentration of substrate is at least five times greater than the concentration of binding sites Equation 3 collapses to the more familiar Michaelis–Menten kinetics (blue line in Figure 2). But when the concentration of substrate is equal to the concentration of binding sites, the kinetics look like the orange line in Figure 2. In the case of rubisco, the *K*_*m*_ for RuBP is 50–100 times less than typical RuBP concentrations. In this case the red line in Figure 2 describes the kinetics and shows that the relationship is essentially either‐or. Another way to think of this is that the Michaelis‐Menten equation assumes free substrate while we usually measure free plus bound substrate. When considering the case of CO_2_ binding to activated rubisco we use some measure of free CO_2_ and as a result, CO_2_ kinetics can be modeled by Michaelis‐Menten kinetics.

## 3 Inputs Versus Outputs

Equation 2 assumes that photosynthetic carbon metabolism depends only on inputs; the outputs from the CBB cycle are considered to have an infinite capacity. Unusual behavior of photosynthesis as a function of CO_2_ were noted in the earliest curves of *A* versus CO (Jolliffe and Tregunna 1968)_2_, now routinely called *A/C*_*i*_ curves (von Caemmerer and Farquhar 1981) even though often *A* is plotted versus CO_2_ inside the chloroplast. Progress toward understanding the ceiling that was often observed in *A/C*_*i*_ curves was published by Sharkey (1985). He proposed that at very high rates of photosynthesis, the ability to use the outputs of photosynthetic metabolism could limit *A*. This is known as triose phosphate use limitation, (TPU). This could lead to CO_2_ or oxygen‐insensitive photosynthesis (Sharkey 1985). This behavior was often seen when *A/c*_*i*_ curves were determined in high light, especially when measurements were made at leaf temperatures below growth temperature. This was interpreted to mean that output metabolism, especially sucrose synthesis, is more sensitive to temperature than is input metabolism. But many times it was found that photosynthesis was not just insensitive to CO_2_ or oxygen but was inversely sensitive. Increasing CO_2_ inhibits *A*. The explanation is that because photorespiration consumes fixed carbon, it acts as an end‐product, or output of the CBB cycle. When photosynthesis is limited by end product usage, decreasing photorespiration decreases one of the end products and so decreases *A*; photosynthesis exhibits reverse sensitivity to CO_2_ and O_2_ (Harley and Sharkey 1991).

Further improvements in the evolution of our understanding of photosynthetic carbon metabolism were published by Yin et al. (2021). The output‐limited behavior of photosynthesis, usually called triose phosphate utilization (TPU) limitation is frequently seen at high CO_2_ and high light (and low measuring temperature). This idea was developed more fully, and new equations were derived, by Busch et al. (2018). Two new parameters were introduced: *α_G_*, the proportion of carbon that enters photorespiration but leaves as glycine and *α_S_*, the amount of carbon that leaves as serine. These parameters have different effects on gas exchange behavior; between 0% and 100% of the carbon that first enters photorespiration as phosphoglycolate could possibly leave as glycine but α_S_ can only vary between 0% and 75% of the carbon that enters as phosphoglycolate. A parameter used to describe rubisco affinities for CO_2_ and O_2_, Γ*, is affected when glycine exits the pathway but not when serine exits. Equation 2 then becomes:

(4) $$A=v_{c}-0.5{∙(1-\alpha_{G})∙v}_{o}-R_{L}.$$

**Figure 3:**
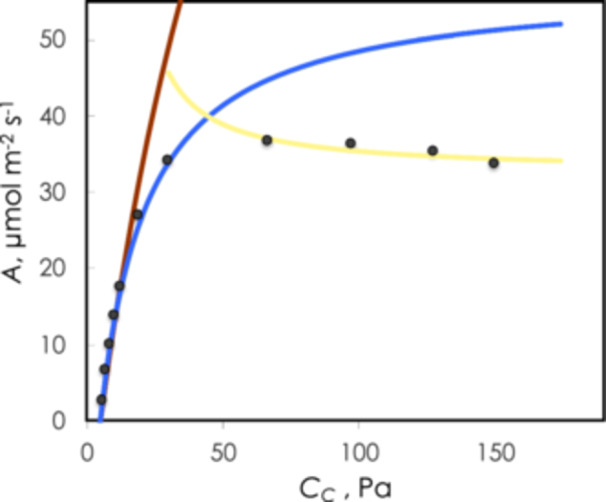
Response of photosynthetic CO_2_ uptake as a function of CO_2_ calculated to be at rubisco. The black circles are the observed rates while the red line is the fitted line assuming RuBP saturated kinetics (rubisco‐limited), the blue line assumes RuBP limited rates, and the yellow line assumed triose phosphate use limitation as described in Equation 5 in the text. The coincidence of RuBP‐saturated and RuBP‐limited rates at low CO_2_ is observed often and represents optimal use of resources. In this case only one data point is in the predicted RuBP‐limited condition. [Color figure can be viewed at wileyonlinelibrary.com]

Equation 4 can be expanded to fit gas exchange behavior when photosynthesis is limited by the formation of end products. So, *A*_*T*_, the rate of photosynthesis when a leaf is TPU‐limited, can be modeled as (Busch et al. 2018):

(5) $$A_{T}=\left(1-\frac{(1-\alpha_{G})∙\Gamma^{*}}{C_{c}}\right)∙\frac{3∙\mathrm{TPU}}{1-0.5∙(1+3\alpha_{G}+4\alpha_{S})∙2∙(1-\alpha_{G})∙\Gamma^{*}/C_{c}}-R_{L}.$$

where *C*_*c*_ is CO_2_ partial pressure at rubisco. This equation will predict how photosynthesis will respond to CO_2_ when photosynthesis is TPU limited. The effect of this on photosynthetic rate is shown in Figure 3 (yellow line). Fu et al. (2023) used isotopically nonstationary metabolic flux analysis (MFA) to estimate that it is common for serine export to reach 40% of the carbon that enters photorespiration while glycine export is generally much less than this amount.

Busch et al. (2018) connected this carbon loss to the accompanying loss of amino groups and have provided an in‐depth treatment of the modeling of the combined carbon and nitrogen metabolism. Using Equation 4 and allowing for diffusion effects allows excellent agreement between gas exchange behavior and underlying biochemistry (Figure 3).

While the transition from rubisco to RuBP regeneration is abrupt, the transition to *TPU*‐limited has not been studied in as much detail but it appears that it is not as abrupt.

## 4 Respiration in the Light

While there have been significant improvements in understanding of photorespiration and its effects on gas exchange behavior, there is less to report on the parameter *R*_*L*_ or *R*_*d*_. Initially called dark respiration in the light, Farquhar et al. (1980) wrote that “For want of a better term we call this dark respiration” and assumed it was mitochondrial in origin. von Caemmerer and Farquhar (1981) called the parameter “day respiration—CO_2_ evolved other than through the photorespiratory pathway.” Calling it day respiration allowed the notation in Equation 2 to stay the same but, because it is not daytime‐dependent but light‐dependent, we use *R*_*L*_ – CO_2_ evolved in the light other than through the photorespiratory pathway. Generally, *R*_*L*_ is less than respiration in the dark (Gong et al. 2018). Measuring *R*_*L*_ is difficult (Yin and Amthor 2024). Methods include measuring light response at very low light [Kok method and its successor, the Yin method (Schmiege et al. 2024)] or measurement at low CO_2_, [the Laisk method (Schmiege et al. 2024)]. Both methods measure at low rates of photosynthesis, often below the compensation point. Methods based on isotopic disequilibrium have also been reported (Gong et al. 2018). These methods have the advantage that they can be done at physiological rates of photosynthesis. Isotopic methods can be used to estimate *R*_*L*_ in forests (Heskel and Tang 2018).

## 5 Origin of the Carbon Lost in *R*_*L*_

The origin of *R*_*L*_ has been the subject of much speculation [reviewed by Tcherkez et al. (2017)]. The electron transport part of mitochondrial metabolism appears to play a significant role in photosynthesis while carbon metabolism is limited to the mitochondrial role in photorespiration (Scheibe 2019). When leaves are fed ^13^CO_2_, TCA cycle intermediates are always labeled less than 5%, sometimes much less (Calvin and Massini 1952; Szecowka et al. 2013; Ma et al. 2014; Xu et al. 2022; Fu et al. 2023). Nevertheless, it was clear from fitting gas exchange data that more CO_2_ is released than can be accounted for by photorespiration. There must be an additional source, or sources, of CO_2_ release during photosynthesis of sufficient magnitude to affect gas exchange behavior, but little to none of this carbon comes from TCA cycle reactions.

There are many processes that release CO_2_ that likely are active in the light (Tcherkez et al. 2017; Yin and Amthor 2024). These include fatty acid synthesis (CO_2_ released by pyruvate decarboxylation), and methylerythritol 4‐phosphate pathway for isoprenoid synthesis, (CO_2_ released by 1‐deoxy‐d‐xylulose‐5‐phosphate synthase). Another process that could result in CO_2_ loss in the light is the oxidative pentose phosphate pathway (OPPP), either inside or outside on the chloroplast. *We propose that the OPPP in the cytosol of plant cells is responsible for most of the CO*_*2*_ *efflux identified as R*_*L*_.

The OPP pathway involves glucose‐6‐phosphate dehydrogenase (G6PDH), which oxidizes G6P. Eventually, CO₂, two NADPHs, and ribulose 5‐phosphate (Ru5P) are produced. In our scheme the Ru5P re‐enters the chloroplast via the xylulose 5‐phosphate/phosphate transporter, bypassing part of the CBB cycle and supplying NADPH to the cytosol for biosynthetic reactions. We propose specifically that it is the OPP pathway that originates outside of the chloroplasts but ends with import of a pentose phosphate into the chloroplasts. Below we discuss biochemistry underlying this photosynthetic CO_2_ exchange and show how a cytosolic OPP pathway explains several anomalies in the behavior of gas exchange of photosynthesis.

## 6 Gas Exchange Anomalies Explained by OPP Pathway Accounting for Most of *R*_*L*_

The Calvin–Benson–Bassham (CBB) cycle is central to photosynthetic carbon assimilation. Recent studies using ^13^C labeling have uncovered anomalies in the labeling patterns of CBB intermediates, prompting further examination into the underlying metabolic processes. Here, we review key findings that can explain these anomalies.

- *lack of complete labeling in CBB intermediates*
- *overabundance of fully unlabeled molecules*
- *three phases in labeling kinetics of CBB intermediates*

### 6.1 Lack of Complete Labeling in CBB Intermediates

One of the more puzzling observations in ^13^C labeling studies of the CBB cycle is the incomplete labeling of its intermediates. While CBB cycle intermediates label to 80%–90% ^13^C within a very few minutes, the remaining 10%–20% of labeling occurs at a much slower rate (Mahon et al. 1974; Hasunuma et al. 2010; Nägele et al. 2010; Szecowka et al. 2013; Ma et al. 2014). Early studies hypothesized the existence of metabolically inactive pools to explain this slow labeling phase (Hasunuma et al. 2010; Szecowka et al. 2013; Ma et al. 2014).

**Figure 4:**
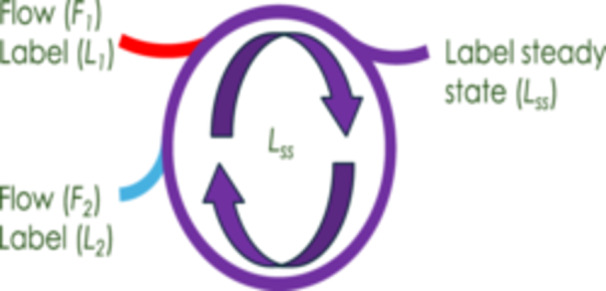
Schematic showing how two inputs will result in a steady‐state label different from either input. *F = Flow, L *= degree of label. When applied to the Calvin Benson Bassham cycle and the data reported here *F*_*1*_ = net rate of CO_2_ assimilation, *A*, *L1* = 100% ^13^CO_2_, *F*_*2*_ = rate of the glucose 6‐phosphate shunt, *L2* = degree of label in cytosolic glucose 6‐phosphate, *L*_*ss*_ = degree of label in Calvin–Benson–Bassham intermediates. [Color figure can be viewed at wileyonlinelibrary.com]

**Figure 5:**
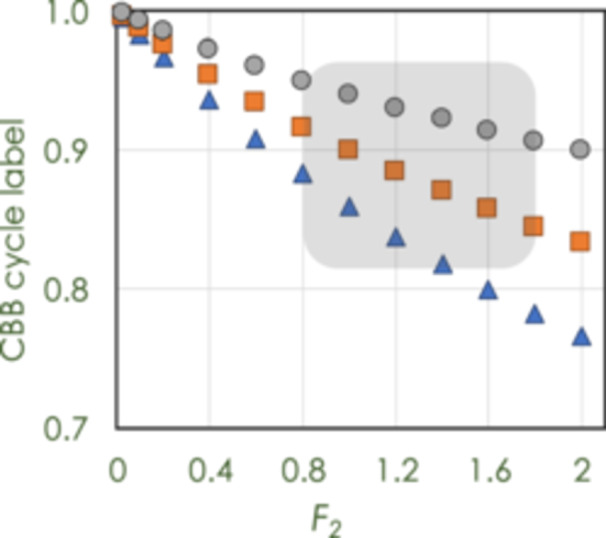
The degree of label as a function of the hypothetical rate of respiration in the light, *R*_*L*_. The gray highlighted region shows the typical range of *R*_*L*_ (*F*_*2*_) and *L*_*2*_, label of carbon in the cytosolic glucose 6‐phosphate pool. The gray circles are outputs of Equation 6 assuming the cytosolic G6P is 90% ^13^C, the orange squares assume 50%, and blue diamonds assume 30%. This large range of assumptions result in an 82% to 95% degree of label in Calvin‐Benson‐Bassham cycle intermediates, the typical range that has been reported. [Color figure can be viewed at wileyonlinelibrary.com]

However, biochemical evidence for such pools is lacking. An alternative explanation is that unlabeled carbon can move from the cytosol, through the cytosolic OPP pathway, and into the chloroplast. Recent MFA studies have provided support for this hypothesis (Xu et al. 2022). This can be modeled assuming two inputs into a common pool of carbon (Figure 4). The label can be described by the following equation:

(6) $$L_{\mathrm{ss}}=\frac{F_{1}∙L_{1}+F_{2}∙5{∙L}_{2}}{F_{1}+F_{2}∙5}$$

where *L*_*ss*_ is the steady‐state label, *F*_*1*_ is the flow rate of CO_2_ (assimilation), *L*_*1*_ is the degree of label (in this case label in the ^13^CO_2_ and set to 100%), *F*_*2*_ is the flow rate through the cytosolic OPP pathway (a range of values as seen in estimates of *R*_*L*_). The denominator is the total flow. Based on other evidence given below we assume that *F*_*2*_ is a flow of carbon from the cytosolic OPPP and so is multiplied by 5 to account for the five carbons that enter the chloroplast as Xu5P, diluting the ¹³C label in proportion to its unlabeled carbon content. Equation 6 was used to construct Figure 5. Figure 5 shows that, reasonable values for fluxes (*R*_*L*_, red box) and labels (orange box) overlap at very reasonable values of presumed input of relatively unlabeled carbon into the CBB cycle.

**Figure 6:**
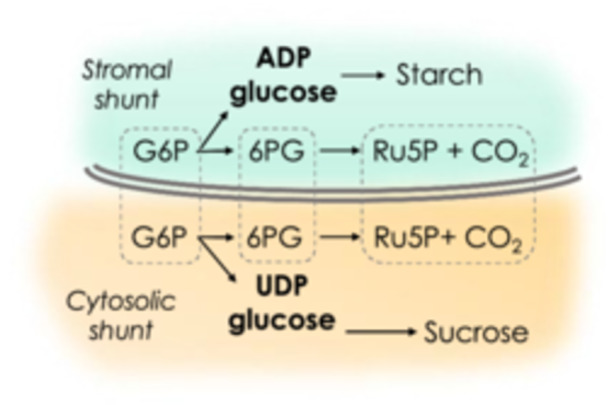
Scheme for using ADPG and UDPG to report the degree of label in the stromal or cytosol. [Color figure can be viewed at wileyonlinelibrary.com]

The glucose 6‐phosphate (G6P) in the stroma is highly enriched with ^13^C as determined by measuring ADP‐glucose which is derived from stromal G6P (Xu, Koroma, et al. 2024). The labeling of ADPglucose (chloroplast reporter) and UDPglucose (cytosol reporter), as shown in Figure 6, confirms that the oxidative pentose phosphate pathway flux is normally entirely cytosolic) (Xu et al. 2022). The stromal OPP pathway cannot account for the lack of complete labeling since the G6P in the stroma would not be depleted of ^13^CO_2_. The cytosolic OPP pathway can easily account for the lack of complete labeling of the CBB cycle intermediates.

### 6.2 Overabundance of Fully Unlabeled Molecules

**Figure 7:**
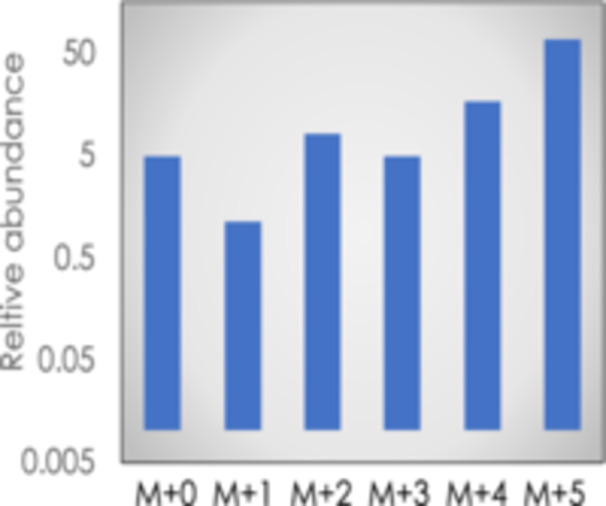
Relative abundance of isotopologues when the degree of label of pentose 5‐phosphate was 70.1%. Data from (Xu et al. 2022). [Color figure can be viewed at wileyonlinelibrary.com]

Another interesting observation is the overabundance of fully unlabeled molecules (M + 0 isotopologues) in CBB intermediates, even when the average degree of label of the metabolite is 70% to 90% (Hasunuma et al. 2010; Sharkey et al. 2020). The notably higher abundance of M + 0 compared to M + 1 (Figure 7), suggests the presence of a carbon source containing five carbon atoms covalently bound together that are entirely unlabeled. Sharkey et al. (2020) proposed this results from carbon flux from significantly unlabeled free glucose through the cytosolic OPP pathway. This makes a G6P shunt bypassing the non‐oxidative pentose phosphate pathway reactions of the CBB cycle. The fully unlabeled molecules follow the OPP pathway and then enter the chloroplast through the xylulose 5‐phosphate/phosphate transporter (Eicks et al. 2002; Hilgers et al. 2018) and possibly other transporters that can transport pentose phosphates.

An influx of fully unlabeled pentose phosphates at 2% of the carboxylation rate would cap maximum labeling at 90%, thus explaining the presence of more M + 0 ions than expected (Allen and Young 2020). By testing multiple MFA models with different unlabeled carbon sources Xu et al. (2022) found that the overabundance of M + 0 isotopologues is best explained by the slow turnover of cytosolic and vacuolar sugars, which slowly feed unlabeled glucose and fructose into the CBB cycle through the G6P shunt over time. The model created by Xu et al. (2022) includes these processes and explains the high levels of M + 0 isotopologues observed in CBB cycle intermediates (Xu et al. 2022). This finding matches previous studies that emphasized the role of vacuolar invertase and sucrose transporters in the slow turnover of vacuolar sugars (Nagle and Morowitz 1978; Uys et al. 2007; W. Patrick et al. 2013)).

### 6.3 Three Phases in Labeling Kinetics of CBB Intermediates

**Figure 8:**
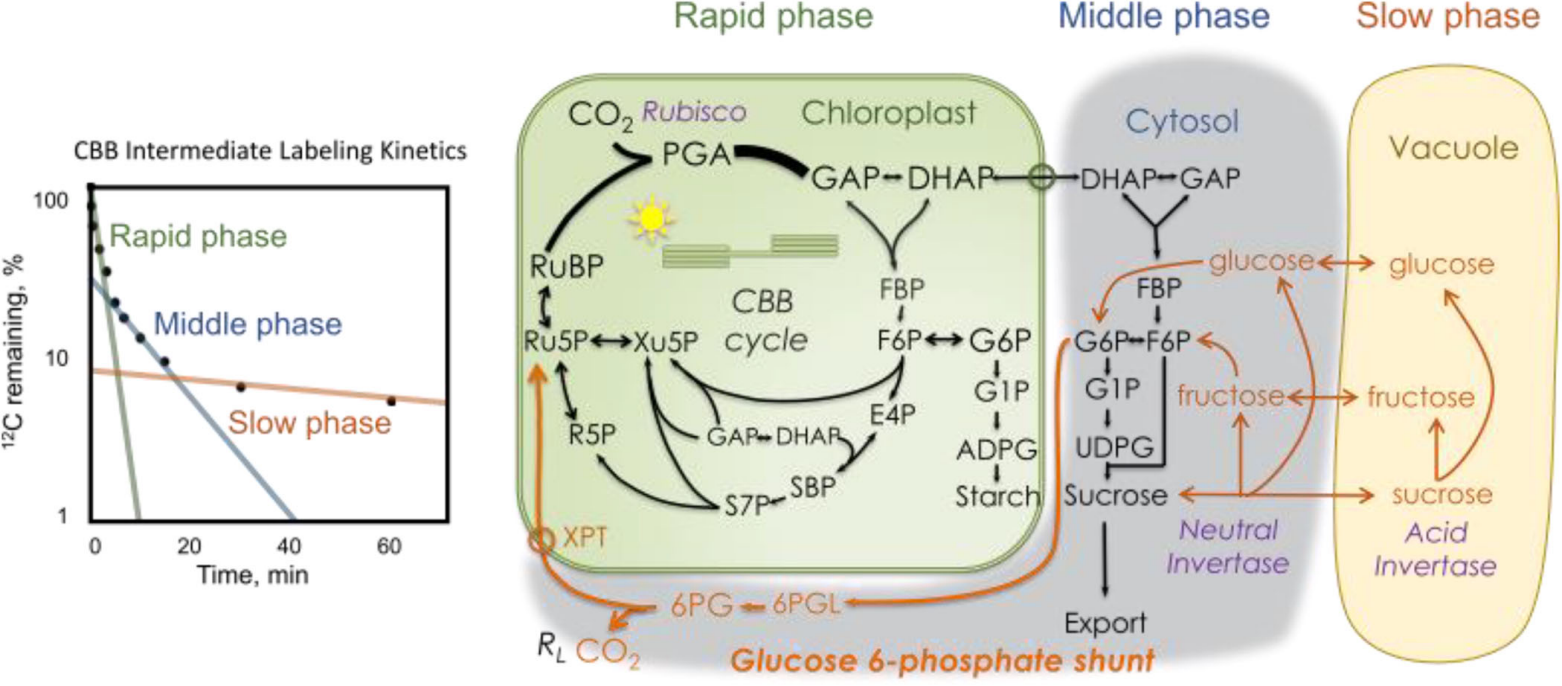
Pathways contributing to incomplete labeling of the Calvin‐Benson‐Bassham cycle intermediates. Orange lines show the path of carbon contributing relatively unlabeled carbon to the Calvin–Benson–Bassham cycle. RuBP = ribulose 1,5‐bisphosphate, PGA = 3‐phosphoglycerate, DHAP = dihydroxyacetone phosphate, GAP = glyceraldehyde 3‐phosphate, S7P = sedoheptulose 7‐phosphate, SBP = sedoheptulose bisphosphate, Ru5P = ribulose 6‐phosphate, R5P = ribose 5‐phosphate, Xu5P = xylulose 5‐phosphate. (redrawn from Xu et al. 2022). [Color figure can be viewed at wileyonlinelibrary.com]

Previous studies have shown that CBB cycle intermediates exhibit rapid initial labeling, followed by a slower progression toward full labeling (Hasunuma et al. 2010; Nägele et al. 2010; Szecowka et al. 2013; Ma et al. 2014). Xu et al. (2022) further showed that extending the labeling time to 2 h uncovered three distinct phases—rapid, middle, and slow—in the labeling kinetics of CBB cycle intermediates, each matching a different metabolic process (Figure 8). Using a triexponential model, Xu et al. (2022) hypothesized these phases to be: (1) rapid labeling due to the fixation of incoming ^13^CO_2_), (2) slower labeling due to the dilution by weakly labeled carbon from cytosolic glucose re‐entering the CBB cycle via the G6P shunt; and (3) very slow labeling due to the further dilution by unlabeled carbon from vacuolar sugars (Xu et al. 2021). The fact that ADPG labeled the same as CBB cycle intermediates rules out starch or other plastid pool as the source of unlabeled carbon. This model, supported by nonlinear regression and statistical model selection criteria, eliminates the need to invoke metabolically inactive pools and provides a more comprehensive understanding of carbon flow in photosynthetic cells (Uys et al. 2007; Xu et al. 2021). The inclusion of cytosolic and vacuolar sugar pools in the metabolic network removes the need for metabolically inactive pools and explains the prolonged dilution of ^13^C label, as these pools act as reservoirs of unlabeled carbon that slowly re‐enter the CBB cycle. The combination of cytosolic and vacuolar sugar pools into the metabolic network of the CBB cycle offers a more complete picture of carbon flow in photosynthetic cells. The three distinct phases of labeling kinetics, the lack of complete labeling, and the overabundance of M + 0 isotopologues are all explained by the re‐entry of unlabeled carbon from these pools into the CBB cycle intermediates. Future studies could further improve this model by exploring the role of other metabolic pathways and environmental factors in shaping the labeling patterns of CBB intermediates.

## 7 Major Source of Respiration in the Light (*R*_*L*_)

Using ^13^CO_2_ isotopic labeling and isotopically nonstationary metabolic flux analysis (INST‐MFA) Xu et al. (2021) suggested that the cytosolic G6P shunt accounts for over 93% of the CO_2_ released as *R*_*L*_. To test alternative ideas, the authors forced the MFA model to attribute *R*_*L*_ to potential sources in the TCA cycle, fatty acid synthesis, or the stromal G6P shunt. When *R*_*L*_ was forced to be explained by the TCA cycle, the sum of squared residuals (SSR, a measure of goodness of fit) increased by over 53%, indicating a poor fit to the experimental ^13^C labeling data. Forcing *R*_*L*_ to be explained by fatty acid synthesis also resulted in a worse fit, with SSR increasing compared to the unconstrained model. The minor contribution of the TCA cycle to *R*_*L*_ may be due to the fact that TCA cycle intermediates exhibited minimal labeling ( < 5%) in hours, consistent with ^13^CO_2_ isotopic labeling studies in Arabidopsis (Szecowka et al. 2013; Ma et al. 2014; Arrivault et al. 2017) camelina (Xu et al. 2021; Xu et al. 2022), and tobacco (Fu et al. 2023).

Xu, Schmiege, et al. (2024) used deuterium labeling in camelina leaves and found that TCA cycle intermediate pools exhibit notably slow turnover over 24 h. This low labeling likely results from large, slowly‐turning over vacuolar organic acid pools, combined with limited flux through active cytosolic and mitochondrial pools of these TCA intermediates during photosynthesis. These findings do not conflict with previous observations on the importance of TCA cycle enzyme activity for maintaining optimal photosynthetic rates but instead show that absolute fluxes through these pathways are too low to explain *R*_*L*_. While enzymes like malate dehydrogenase are essential for the malate valve's function (Nunes‐Nesi et al. 2005; Nunes‐Nesi et al. 2008), quantitative analysis shows they contribute only a small fraction of total energy flux (Walker et al. 2024).

## 8 Dominance of Cytosolic G6P Shunt in *R*_*L*_

The G6P shunt works in both cytosolic and plastidic compartments, though with different activity patterns. The cytosolic G6P shunt functions continuously at relatively steady rates as shown by respiratory loss measurements (Tcherkez et al. 2017; Schmiege et al. 2023). In contrast, the stromal G6P shunt stays mostly inactive during normal daylight conditions due to thioredoxin‐mediated redox control of stromal G6PD (Wenderoth et al. 1997; Hauschild and von Schaewen 2003), and inhibition by its product NADPH (Wakao and Benning 2005; Preiser et al. 2019). A stromal G6P shunt would not result in the lack of complete labeling or the overabundance of M + 0 isotopologues because it would not be introducing unlabeled carbon. The cytosolic G6PDH's are much less regulated (Kruger and von Schaewen 2003; Wakao and Benning 2005) allowing them to remain active in the light.

*R*_*L*_ is mainly driven by cytosolic processes, as shown by isotopic labeling studies. Sharkey et al. (2020) used ^13^CO_2_ labeling in poplar leaves to show that 6‐phosphogluconate (6PG), an intermediate in the OPP pathway, closely matched the labeling of uridine diphosphate glucose (UDPG), a marker for cytosolic G6P when leaves were kept at 30°C. In contrast, adenosine diphosphate glucose (ADPG), reflecting stromal G6P, shows significantly higher labeling, indicating minimal stromal shunt activity under normal conditions. Xu, Schmiege, et al. (2024) used ^13^CO_2_ labeling in tobacco and found that 6PG labeling closely matched UDPG but was lower than ADPG. This pattern persisted across low, ambient, and high CO_2_ concentrations, confirming the cytosolic shunt as the primary source of *R*_*L*_. The cytosolic shunt introduces unlabeled carbon into photosynthesis via hexokinase‐mediated phosphorylation of free glucose, diluting ^13^C in downstream metabolites. This process is supported by the slow turnover of cytosolic and vacuolar sugars, which act as reservoirs of unlabeled carbon (Xu et al. 2022). The lack of stromal shunt activity is due to redox control of stromal G6PD, which is inhibited in the light (Scheibe et al. 1989; Preiser et al. 2019). Xu et al. (2022) also tested a model including both cytosolic and plastidic G6P shunt but found zero fitted flux through the plastidic G6P shunt, showing no significant contribution from this pathway. These findings highlight the cytosolic G6P shunt as the dominant contributor to *R*_*L*_, providing a stable mechanism for CO_2_ release during photosynthesis, independent of CO_2_ levels.

In an early paper we devised a mechanism involving starch cycling (Sharkey and Weise 2016) to explain the lack of complete labeling. This would require simultaneous synthesis and breakdown of starch, which is thought to occur but mainly at the end of the day or when photosynthetic rates are low (Fernandez et al. 2017; Ishihara et al. 2022). However, even if old carbon (unlabeled) could be liberated inside the chloroplast it would have to pass through ADPGlc and so the labeling of ADPG and UDPG rule out a starch‐based mechanism.

## 9 Stability of *R*_*L*_ Across Different CO_2_ Concentrations and Light Intensities

*R*_*L*_ has been shown to stay remarkably stable across varying CO_2_ concentrations. In the study by (Schmiege et al. 2023) the stability of *R*_*L*_ under varying environmental conditions was examined using both traditional steady‐state methods and a new non‐steady‐state dynamic assimilation technique (DAT). The authors found that *R*_*L*_ stayed relatively stable across different CO₂ treatments in paper birch. This stability was consistent using either dynamic assimilation technique (DAT) or steady‐state methods, suggesting that *R*_*L*_ is a robust parameter that does not significantly adapt to short‐term environmental changes in this species. While *R*_*L*_ stayed stable, *C*_*i*_ *∗* (linked to *Γ∗*, the rubisco CO₂ compensation point) was consistently higher with DAT, particularly under high or low CO₂ concentrations, suggesting dynamic shifts in photorespiratory processes, potentially due to glycine export. These findings are inconsistent with the interpretation that *Γ∗*, or more precisely *Γ∗* •(1‐α_g_) accurately reflects rubisco properties since these should not change over short time periods. On the other hand they highlight the importance of *R*_*L*_ as a stable component of plant carbon balance across different species and environments (Schmiege et al. 2023).

Similarly, another study by Xu, Schmiege, et al. (2024) has found that *R*_*L*_ is largely unaffected by changes in CO_2_ levels measured by the Yin method (Yin et al. 2009; Yin et al. 2011), whether low (250 ppm), ambient (450 ppm), or high (1500 ppm). This stability suggests that the metabolic processes contributing to *R*_*L*_, particularly the cytosolic G6P shunt, work independently of CO_2_ availability. The consistency of *R*_*L*_ across CO_2_ concentrations matches the observation that cytosolic G6P levels are tightly controlled and stay relatively constant throughout the day, dropping only at night (Dietz 1985; Gerhardt et al. 1987). This control ensures a steady flow of unlabeled carbon into the photosynthetic system, even as the rate of labeled carbon fixation rises with higher CO_2_ levels.

In a recent study by (Xu et al. 2025), the stability of *R*_*L*_ under high light and high CO₂ conditions was examined using INST‐MFA in camelina. Instead of traditional *R*_*L*_ methods like Kok method (Kok 1948) or Yin method (Yin et al. 2009, 2011), INST‐MFA was used to measure *R*_*L*_ because conventional approaches require measurements near the CO₂ compensation point, risking issues like rubisco deactivation and failing to capture dynamic metabolic changes under high light and high CO₂. Despite significant changes in photosynthetic rates and metabolic fluxes under high light and high CO₂, *R*_*L*_ stayed stable. This stability was mainly attributed to the OPPP, which accounted for 88.6% and 78.0% of *R*_*L*_ in control and high light and high CO₂ conditions, respectively. These findings suggest that *R*_*L*_ is a robust component of plant carbon metabolism, emphasizing its resilience to environmental changes. This stability highlights the importance of *R*_*L*_ in maintaining metabolic balance and further supports the idea that *R*_*L*_ is primarily driven by cytosolic processes, rather than being significantly influenced by changes in photosynthetic rates or carbon partitioning (Xu et al. 2025).

## 10 Heat Stress Shifts *R*_*L*_ Dominance From Cytosol to Plastid

It has been observed that *R*_*L*_ stays stable across different CO_2_ levels and light intensities (Schmiege et al. 2023; Xu, Koroma, et al. 2024; Xu et al. 2025) and 6PG labeling closely matches UDPG but not ADPG. This confirms that the cytosolic G6P shunt is the main source of *R*_*L*_ across varying CO_2_ levels. However, Sharkey et al. (2020) found that when measuring at 40°C instead of 30°C leaf temperature, the main source of *R*_*L*_ shifts from the cytosol to the plastid. At 40°C, the authors observed that 6PG labeling was much higher than UDPG and closer to ADPG, unlike control conditions where 6PG and UDPG labeling were similar. This change shows that the plastidial G6P shunt is turned on during heat stress, changing carbon flow between the OPPP and the CBB cycle. The rates of the cytosolic versus plastid pathway could not be determined. Previous studies showed that heat stress causes the production of hydrogen peroxide (H_2_O_2_) in plant cells, which acts as a signal to turn on antioxidant defenses and reduce oxidative damage, helping plants adjust to high temperatures (Apel and Hirt 2004; Suzuki and Mittler 2006). Preiser et al. (2019) found that under stress conditions like heat or high light, H_2_O_2_ activates plastidial G6PDH, allowing it to function in the OPPP to maintain redox balance and support metabolic flexibility. This highlights the distinct roles of plastidial and cytosolic G6PDH forms, with plastidial versions playing a key role in stress adaptation by controlling carbon flow between the CBB and OPPP, ensuring metabolic and redox stability under environmental stress. Further research is needed to fully understand the mechanisms regulating the shift in *R*_*L*_ dominance from the cytosol to the plastid under stress conditions and its impact on carbon flow and metabolic balance.

## 11 Conclusion

*R*_*L*_ represents a longstanding yet underappreciated component of photosynthetic metabolism. While historically regarded as mitochondrial “dark respiration” occurring during illumination, recent advances in isotopic labeling and metabolic flux analysis have identified the cytosolic G6P shunt via the OPPP as the predominant source of RL under normal physiological conditions. This cytosolic G6P shunt explains anomalous ¹³C‐labeling patterns in the CBB cycle—including incomplete labeling, overabundance of M₀ isotopologues, and multiphase kinetics.

*R*_*L*_ remains remarkably stable across a range of CO₂ concentrations and light intensities, highlighting its constitutive role in maintaining redox balance (via NADPH generation) and supporting carbon flow during photosynthesis. However, under heat stress, the plastidial OPPP becomes the primary contributor to *R*_*L*_, likely due to redox (especially H_2_O_2_) activation of stromal G6PDH. This dynamic compartmentation underscores *R*_*L*_'s role in coordinating carbon and redox metabolism.

Together, these findings support a new view of *R*_*L*_ as a regulated and compartmentalized metabolic shunt—rather than residual byproduct of mitochondrial respiration. This new perspective enhances our understanding of plant carbon balance and improves the interpretation of gas exchange measurements. Future work should further dissect *R*_*L*_'s regulation and integration into broader metabolic and stress‐response networks. A deeper mechanistic understanding of *R*_*L*_ may inform strategies for improving photosynthetic efficiency and stress resilience in plants.

## Conflicts of Interest

The authors declare no conflicts of interest.

## AI

ChatGPT was used to edit the abstract and introduction to improve clarity and readability. Other sections were not subjected to ChatGPT.

## Data Availability

The authors have nothing to report.
